# Precipitation in a warming world: Assessing projected hydro-climate changes in California and other Mediterranean climate regions

**DOI:** 10.1038/s41598-017-11285-y

**Published:** 2017-09-07

**Authors:** Suraj D. Polade, Alexander Gershunov, Daniel R. Cayan, Michael D. Dettinger, David W. Pierce

**Affiliations:** 1 Climate, Atmospheric Science and Physical Oceanography (CASPO), Scripps Institution of Oceanography, La Jolla, CA USA; 2 United States Geologic Survey, Carson City, Nevada USA

## Abstract

In most Mediterranean climate (MedClim) regions around the world, global climate models (GCMs) consistently project drier futures. In California, however, projections of changes in annual precipitation are inconsistent. Analysis of daily precipitation in 30 GCMs reveals patterns in projected hydrometeorology over each of the five MedClm regions globally and helps disentangle their causes. MedClim regions, except California, are expected to dry via decreased frequency of winter precipitation. Frequencies of extreme precipitation, however, are projected to increase over the two MedClim regions of the Northern Hemisphere where projected warming is strongest. The increase in heavy and extreme precipitation is particularly robust over California, where it is only partially offset by projected decreases in low-medium intensity precipitation. Over the Mediterranean Basin, however, losses from decreasing frequency of low-medium-intensity precipitation are projected to dominate gains from intensifying projected extreme precipitation. MedClim regions are projected to become more sub-tropical, i.e. made dryer via pole-ward expanding subtropical subsidence. California’s more nuanced hydrological future reflects a precarious balance between the expanding subtropical high from the south and the south-eastward extending Aleutian low from the north-west. These dynamical mechanisms and thermodynamic moistening of the warming atmosphere result in increased horizontal water vapor transport, bolstering extreme precipitation events.

## Introduction

Mediterranean climates represent a precarious balance, being exposed to mid-latitude weather in winter, while chronically challenged by subtropical dryness in summer. Characterized by typically infrequent frontal storms confined to the cold season, water resources in Mediterranean climates are volatile from year to year due to sample variance, i.e. one large storm can make an inordinate difference to total annual precipitation^1, 2^. Globally, CMIP5 climate models consistently project future drying in subtropical regions but increasing precipitation over the mid and high latitudes^3, 4^. How do projections play out over Mediterranean climate regions characterized seasonally by subtropical and mid-latitude influences?

For most Mediterranean climate (MedClim) regions around the globe, the models consistently project drier futures^5–7^. MedClim regions, receiving most of their precipitation during winter, are semiarid so that moderate declines in the hydroclimate can engender substantial socioeconomic impacts. However, over California the model consensus is poor, with some models trending drier, others – wetter^5^. For the peak of the wet season (December – February), as we shall see below, a weak trend towards wetter conditions in California is projected.

Recently, a number of studies have explored causes of projected precipitation change over California. For example, ref. 8 attributed the trend towards wetter winters over California to a stronger sub-tropical jet stream. Analysis by ref. 9 found that model-to-model differences in winter precipitation over California are mainly explained by differences in projected mid-latitude cyclone activity. ref. 10 explained trends towards wetter California by a lengthening of the intermediate-scale stationary waves in the presence of accelerated sub-tropical westerlies. These previous studies of future precipitation primarily resolved annual to seasonal time scales, focused on California alone, and mainly investigated dynamical mechanisms.

As we shall see, compared to other MedClim regions, seasonal precipitation projections over California are characterized by uncertainty. This uncertainty stems in part from two opposing and robust signals: decreased frequency and increased intensity of projected daily precipitation^5^. The goal of this work is to investigate projected precipitation changes over California by exploring the similarities and differences in daily precipitation projections over all MedClim regions. We study changes in the statistics of daily precipitation, with a focus on extreme events, to understand projected changes in precipitation regimes over MedClim regions and their sensitivity to regional thermodynamic and dynamic processes. In particular, we aim to achieve a clearer understanding of projected precipitation over California than is implied by the large model spread in annual precipitation.

## Results

### Definitions

This study focuses on late-21^st^ Century *winter* (Northern Hemisphere–NH: December-January-February or DJF; Southern Hemisphere–SH: June-July-August or JJA) precipitation changes relative to the late 20^th^ Century in five Mediterranean regions around the globe – the Mediterranean basin (MED), South America (SAA), California (CAL), South Africa (SAF), and Australia (AUS). Winter season is the peak of the wet season over these MedClim regions. Wintertime contribution to total precipitation by region is as follows: MED: 38%; CAL: 57%; SAA: 50%; SAF: 40%, AUS: 38% (Fig. S1a). The definition of MedClim regions is taken from ref. 11 and provided in the Methods Section. The SH MedClim regions are less spatially extensive than their NH counterparts. We must be mindful of this fact when interpreting results, particularly for SAF, which is represented by as few as a couple of grid cells in the native resolution of the coarsest GCMs. All models were re-gridded to the common 2 × 2 grid, however, same as the observations, prior to any analysis. To ameliorate the well-known GCM drizzle problem^12^, all daily precipitation values <1 mm were assigned zero values. A 30-member set of the Coupled Model Intercomparison Project, version 5 (CMIP5) GCMs was employed. Future projections for *late* 21^*st*^ *century* (2060–2089) based on the Representative Concentration Pathway 8.5 (RCP 8.5) scenario were then compared to the historical *base period* of 1960–1989. Please see the Methods section for further details.

### Changes in total winter precipitation

**Figure 1:**
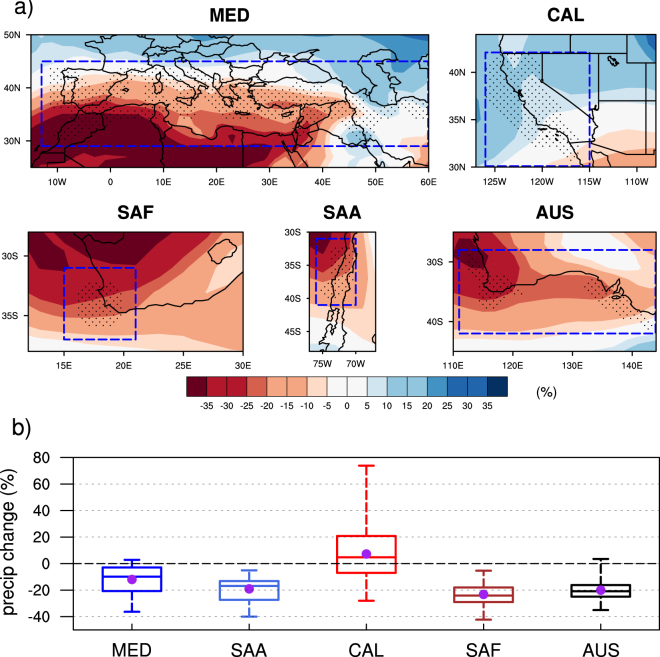
CMIP5 winter precipitation changes for (**a**) multi-model ensemble mean, (**b**) area average over the Mediterranean-climate grids (stippled region shown in Fig. 1a) by 2060–2089, relative to the historical period 1960–1989, using the RCP8.5 forcing scenario over the five global Mediterranean-climate regions. Stippling indicates the grid cells that satisfy the Mediterranean climate criteria and dashed boxes indicate its’ maximum regional extent. The multi-model ensemble average shown by the circle and the median indicated by the horizontal line, the box the interquartile range, and whiskers the range containing 99% of projections. Figure was plotted using NCAR Command Language Version 6.3.0 (NCL; ref. 31).

The projected late-century changes in ensemble mean winter precipitation are expressed in percent of historical (1960–89) precipitation and shown in Fig. 1a. Except over CAL, the models consistently project future drying over MedClim regions, especially over those in the SH. In composite, all of the MedClim regions exhibit a meridional gradient of precipitation change, with less drying at higher vs subtropical latititudes. In CAL, the ensemble average winter precipitation decreases in the south, especially over northern Baja California, Mexico, by as much as 10%, and increases over central-northern California by 5 to 15%. CAL is projected to become wetter in winter aggregate, albeit model consensus is weak.

Projected average precipitation changes in each region (averaged only over grid cells that satisfy the MedClim criteria: see Methods Section for details) are summarized in Fig. 1b. Over the four MedClim regions other than CAL, nearly all of the models consistently project drying. Over CAL, two-thirds of the models project wetter winters. Of course, there are significant model-to-model differences in the magnitude of the projected change. On average, CMIP5 models project a 12% reduction (ranging from 1% to −38%) in precipitation over MED, a 19% reduction (ranging from −5% to −40%) over SAA, a 23% reduction (ranging from −5% to −42%) over SAF, and a 20% reduction (ranging from 1% to −38%) over AUS. MED, SAA, SAF and AUS all exhibit significant model agreement in the sign of changes, with a strong majority of models indicating drying, and a comparable magnitude of model spread (ranging from 35 to 39%). Over CAL, the mean precipitation is projected to increase by 7% (ranging from −28% to 78%). Even though the projected mean tendency over CAL is towards wetter winters, model spread is three times that over the other regions making CAL stand out as the only MedClim region where climate models strongly disagree about wintertime precipitation change. The definition of MedClim regions does not affect our overall conclusion (Fig. S1b). Moreover, model resolution does not seem to systematically affect model realism in simulating the winter contribution to total precipitation, an essential feature of Mediterranean climates (Fig. S2). Nor does model realism systematically affect the magnitude of projected change or even its sign in the case of CAL (Fig. S3). This is in line with results of ref. 13 who showed that GCM realism vis a vis salient features of the observed regional climate is not a relevant factor in precipitation changes that the GCMs project for the future climate of the Western U.S.

### Changes in daily precipitation frequency and intensity

**Figure 2:**
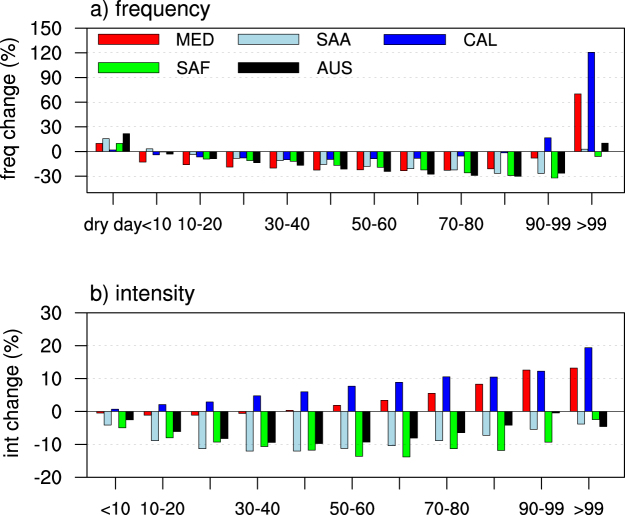
CMIP5 multi-model ensemble average changes in winter precipitation (**a**) frequency and (**b**) intensity average in different percentile categories over the Mediterranean-climate grids (stippled region shown in Fig. 1a) by 2060–2089, relative to the historical period 1960–1989, using the RCP8.5 forcing scenario over the five global Mediterranean-climate regions. Figure was plotted using NCAR Command Language Version 6.3.0 (NCL; ref. 31).

End-of-century changes in the multi-model mean frequency of dry days (<1 mm/day) and days with precipitation in different percentile categories calculated with respect to the historical period are shown in Fig. 2a. Importantly, a projected decrease in the number of winter wet days with precipitation amounts below the 90^th^ percentile threshold is evident in all regions. In conjunction, the frequency of dry days increases in all MedClim regions. However in CAL, the ensemble-average increase in dry days is projected to be quite small (+2 days/winter) compared to that in the other regions (+8 to 17 days/winter). This small overall change in CAL reflects the balance between projected decreases in the frequency of dry days in the north and increases in the south^5^.

On the heavy side of the precipitation distribution, changes in the frequency of days with extreme precipitation (>99^th^ percentile) vary greatly, notably between the NH and SH MedClim regions. Large increases in the frequency of extreme precipitation days are projected over MED and CAL (+68% and +117%, respectively). SH regions, on the other hand, display small and inconsistent trends in the frequency of extreme precipitation (−8% over SAF to +11% over AUS).

The large increase in extreme precipitation frequency over CAL (amounting to more than one extra >99^th^ percentile day each winter by late-century) is consistent with an overall change in the shape of the probability density function (PDF) of daily precipitation amounts. Compared to the other MedClim regions, CAL exhibits smaller decreases in precipitation frequencies for categories from near-median daily precipitation and above, more strongly increased frequency of heavy precipitation (~15% in the 90–99% category), and more-than-doubling in the frequency of extreme precipitation as described above.

Precipitation intensity decreases in all categories in the MedClim regions of the SH but increases in those of the NH (Fig. 2b) except below the 40^th^ percentile threshold over the MED region. Precipitation intensity increases with percentile categories over the MED and CAL regions; however, CAL generally shows a much stronger increase. In the SH, the decrease in precipitation intensity is stronger in the moderate percentile threshold categories compared to the dry and wet tails of the PDF. This suggests that projected precipitation PDFs undergo shape changes amounting to a flattening for the SH MedClim regions in contrast to an extension and thickening of the wet tail for the NH regions, particularly for CAL, where precipitation intensity increases progressively in all intensity categories.

**Figure 3:**
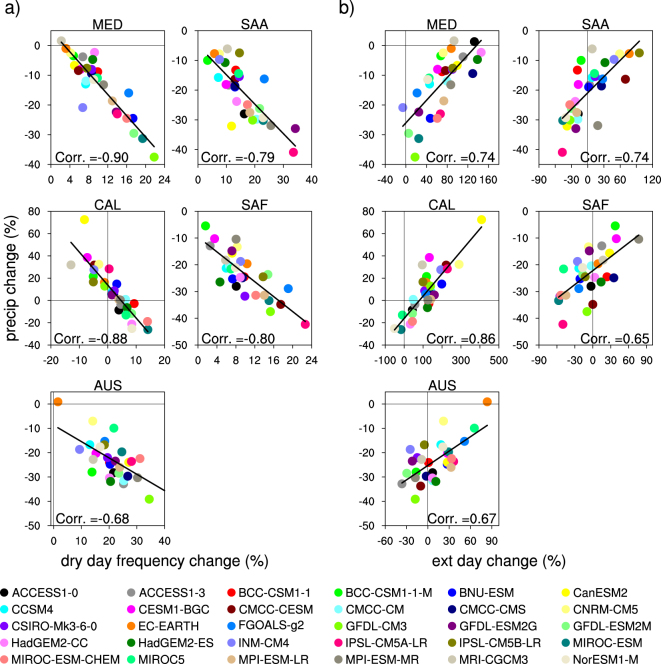
CMIP5 winter precipitation changes versus (**a**) changes in dry day and (**b**) extreme precipitation day frequency average over the Mediterranean-climate grids (stippled region shown in Fig. 1a) by 2060–2089, relative to the historical period 1960–1989, using the RCP8.5 forcing scenario over the five global Mediterranean-climate regions. Black line indicates the regression line (and correlation, Corr) corresponding to all CMIP5 models. Figure was plotted using NCAR Command Language Version 6.3.0 (NCL; ref. 31).

We next consider the individual GCMs to investigate how changes in the frequencies of dry days and days with extreme precipitation might affect changes in total seasonal precipitation change for each MedClim region (Fig. 3). All of the regions exhibit significant and clear inverse relationships between changes in dry day frequency and the changes in total winter precipitation (Fig. 3a). Across the 30 GCMs, the highest correlations (r = ~0.9) occur over MED and CAL with 80% of the inter-model variance in projected seasonal precipitation changes explained by changes in dry day frequency. The slope of the regression between these two quantities (~0.3%) is similar over all of the regions. CAL displays a larger spread in winter precipitation, which is better explained by the changes in dry day frequency compared to other regions. Although the across-model relationships between projected precipitation and dry day frequency is consistent from region to region, the regional changes within individual GCMs are not very consistent; a model with a large increase in dry day frequency over one region does not necessarily associate with a large increase over another.

A rather strong relationship also exists between projected precipitation change in a given model and its change in the frequency of extreme precipitation over all regions (Fig. 3b). Correlations over the 30 GCMs range from 0.65 in SAF to 0.86 in CAL. Overall, the change in winter precipitation is determined by the interplay between changes in frequency of dry days and very heavy precipitation days. Changes in the frequency of dry days are inversely related to changes in frequency of heavy precipitation days (correlations ranging from −027 in AUS to −0.60 in CAL). However, trends in the number of dry days dominantly determine the change in winter precipitation over MED, SAA, and SAF, explaining ~60% of this change (bivariate regression model), whereas trends in extreme precipitation frequency explain ~40% of the seasonal total change. Over CAL and AUS trends in frequency and extremes each explain approximately equal shares of the projected change in winter precipitation.

An example helps to clarify this concept. Over the MED region, although the frequency of extreme precipitation days is clearly projected to increase (Fig. 3b; several models project more than a 100% increase), most models project a drier future. This is due to the greater influence of the projected increased frequency of dry days (Fig. 3a), which translates into decreased frequency in most precipitation intensity categories (Fig. 2a). In CAL, this is also possible, and, HadGEM2-ES provides an example of a GCM projecting increased frequency of dry days and no change in extreme precipitation frequency resulting in a drier future winter precipitation. However, there is a larger model spread in CAL compared to other regions; many models project strong positive trends in the frequency of extreme precipitation, which cause increases in winter precipitation in GCMs marked by weak trends in dry day frequency^14^ but not necessarily in GCMs whose dry day frequency is stronger. BNU-ESM is a model showing a strong increase in the frequency of extreme precipitation (by >200%) and a weak increase in dry day frequency (by 3.8%), and thus an increase in winter precipitation (by 13%).

### Roles of thermodynamic and dynamic processes

Projected precipitation changes over MedClim regions can, to various degrees, be explained by dynamical factors associated with changes in storm tracks and weather regimes, along with thermodynamic factors associated with increased water vapor content in a warmer atmosphere. It is moreover possible that dynamical factors can preferentially affect seasonal precipitation by impacting precipitation frequency^15^, while thermodynamic factors are more likely to influence precipitation intensity, particularly that of extreme events^16^. To provide insights into the relative roles of these dynamic versus thermodynamic influences, we next explore ensemble projected wintertime trends in the surface air temperature (SAT), vertically-Integrated horizontal water Vapor Transport (IVT), and mean sea level pressure (MSLP).

**Figure 4:**
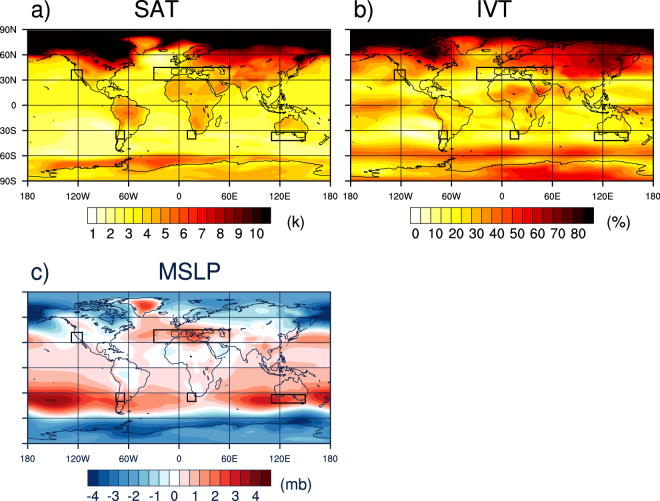
CMIP5 multi-model ensemble average wintertime change in (**a**) surface air temperature; (**b**) mean horizontal integrated water vapor transport (IVT); (**c**) mean sea level pressure. Values are computed over the period 2060–2089, relative to the historical period 1960–1989, using the RCP8.5 forcing scenarios. Black boxes indicate the max latitude and longitude extent of the Mediterranean-climate regions. Figure was plotted using NCAR Command Language Version 6.3.0 (NCL; ref. 31).

Figure 4a shows the ensemble mean projected change in wintertime SAT, which serves as an index of the amount of atmospheric warming and the increased potential to carry moisture. Overall, land areas show greater wintertime warming than oceans. Greenland is a notable exception. The tropical Pacific shows somewhat greater projected warming compared to other ocean areas. On average, SH MedClim regions show less warming (2.1 to 2.3 °C) compared to CAL and MED (3.2 and 3.4 °C, respectively). The pattern of IVT increase (Fig. 4b) generally follows this pattern of increased SAT. ref. 17 shows that this projected IVT trend, averaged zonally, is mainly due to increased water vapor content. This thermodynamic trend makes more moisture available for precipitation, enhancing extremes^16^. SAT and IVT increases are generally larger in the NH than at the same latitudes in the SH, at least partially explaining why increases in heavy to extreme precipitation are larger over the NH MedClim regions than over their SH counterparts.

Regional dynamical circulation trends, in the form of enhanced convergence or divergence, can respectively support or counteract this response to thermodynamic forcing. The ensemble-mean projected change in MSLP is shown in Fig. 4c. Globally, MedClim regions are becoming more subtropical as the subtropical high-pressure belt widens poleward^18^. Over most MedClim regions, the quasi-stationary subtropical high-pressure system is projected to strengthen by end-of-century by 3–6 mb. However, over the North Pacific, the model ensemble projects a deepening of the Aleutian low-pressure system core by ~4 mb and a notable southeastward extension of the Aleutain Low toward CAL (Fig. 4c). ref. 19 suggested that the deepening of the Aleutian Low is related to changes in the mean zonal basic state as well as changes in tropical diabatic heating.

The dynamical tendencies affecting precipitation changes over CAL involve a deeper Aleutian Low to the north and an enhanced subtropical high to the south (Fig. 4b), steering enhanced IVT (40–60%) over CAL (Fig. 4b and c). The strongest instances of these tendencies are associated with atmospheric rivers (ARs) known to cause extreme precipitation^20^ and expected to be bolstered by global warming^17, 21, 22^ via the Clausius-Clapeyron relationship. A long-term increasing trend in land-falling AR activity, as well as winter IVT generally, was recently reported along the west coast of North America in conjunction with observed North Pacific sea surface temperature warming^23^. Notably, the projected increase in IVT over the other MedClim regions, especially those of the SH, is appreciably smaller than that over CAL, ranging from 0 to 20%.

Overall, the SH MedClim regions are projected to moisten the least due to the thermodynamic effect and dynamical factors are projected to steer storms away from these regions. However, both CAL and MED regions are projected to moisten more than their SH counterparts, consistently with the thermodynamic implications of stronger winter warming. Moreover, a southeastward expanding Aleutian Low counteracts the northward expanding Subtropical High over CAL resulting in no appreciable change in dry day frequency, but dynamically promoting land-falling ARs while warming contributes thermodynamically to wetter ARs. The drying trend observed for the other MedClim regions, however, is associated with increased moisture divergence due to the strengthening of quasi-stationary subtropical high-pressure systems, leading to less frequent precipitation in spite of increased moisture-holding capacity of a warming atmosphere.

**Figure 5:**
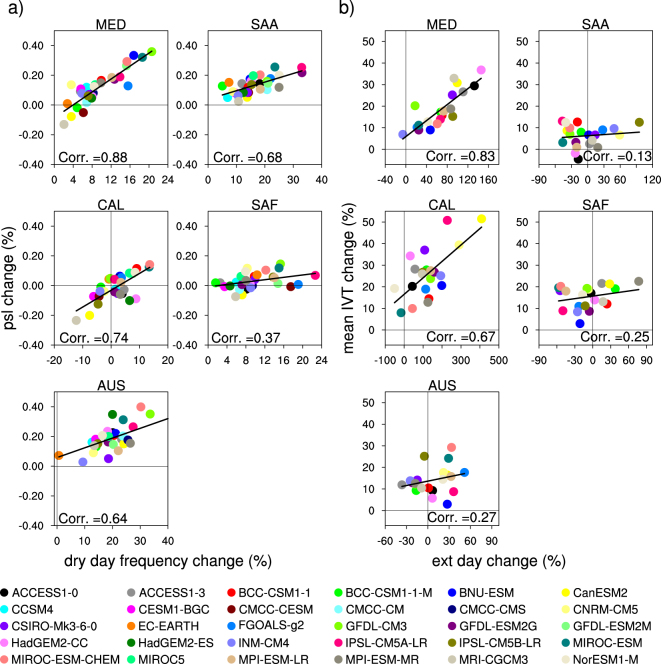
CMIP5 winter trends in (**a**) dry day frequency versus mean sea level pressure and (**b**) very heavy precipitation events versus mean IVT average over the Mediterranean-climate grids (stippled region shown in Fig. 1a) by 2060–2089, relative to the historical period 1960–1989, using the RCP8.5 forcing scenario over the five global Mediterranean-climate regions. Colored dots correspond to specific GCMs. Regression lines and correlation coefficients for each scatterplot are provided on each panel. Figure was plotted using NCAR Command Language Version 6.3.0 (NCL; ref. 31).

Since mean precipitation change is determined by the balance between increased dry day frequency and that of extreme precipitation, we explore whether the inter-model spread in these two quantities is explained by changes in MSLP and IVT, respectively. Figure 5a shows the regional average variation of dry days in response to changes in MSLP for individual models over all MedClim regions. The models generally exhibit positive relationships, i.e., dry days increase with MSLP, although in some regions (notably SAF and CAL), a few models project an increase in dry days with a slight decrease in MSLP. Across the 30 GCMs, a strong correlation exists between MSLP and dry days (r = 0.64 to 0.88) over four of the five regions, while in SAF the correlation is weaker (r = 0.37). The relationship between MSLP and the mean precipitation change is weaker (NH; r ~ 0.56), especially in the Southern Hemisphere (SH; r ~ 0.45) (see online supporting information, Fig. S4), highlighting the direct dynamical link with precipitation frequency.

Similarly, the frequency of extreme precipitation events is strongly linked with IVT over regions where large increases in extremes are projected. The correlation between these two quantities is 0.83 over MED and 0.63 over CAL, whereas it is weak over the SH regions (correlations <0.28). Over SAA, SAF and AUS, increases in IVT are insignificant and the frequency of extreme precipitation events tends to change little on average. This highlights the thermodynamic control on extreme precipitation trends.

## Discussion and Summary

Using daily projections from 30 CMIP5 global climate models forced by the RCP 8.5 scenario during the period of 2060–2089 relative to the period of 1960–1989, we investigated changes in winter precipitation in five MedClim regions around the globe, which are mostly robustly projected to become drier. We emphasized winter precipitation projections over CAL, which are distinguished from the other MedClim regions by greater model spread and, in aggregate, a rather subtle tendency to become wetter. By investigating changes in the frequency and intensity of the daily precipitation distribution, we showed that winter precipitation trends over MedClim regions tend to be the net result of decreasing frequencies of low- and medium-intensity precipitation and, in some regions, increasing trends in extreme precipitation events. Over all the MedClim regions except CAL, decreasing trends in low- and medium-intensity precipitation play the definitive role in causing winter precipitation to decline. In CAL, however, while there is little model consensus on the sign of the overall precipitation frequency trend, GCMs robustly project increased extreme precipitation frequency and intensity. The result for CAL is a weak model ensemble tendency towards wetter winters.

Competing mechanisms operate in determining MedClim precipitation changes. Thermodynamic changes, via enhanced saturation vapor pressure in a warmer atmosphere, are projected to enhance the potential for stronger extreme precipitation events. Changes in large-scale dynamic structures, via poleward extensions of the subtropical high-pressure zone, are expected to steer midlatitude cyclones away from most MedClim regions, i.e. MedClim regions are projected to become more subtropical. In the MED, although dynamics are projected to steer storms away from the region, strong warming results in occasionally stronger extremes due to the thermodynamic effect of enhanced vapor transport in a warmer atmosphere.

California is exceptional. Here projected stronger and more extensive Aleutian Low counteracts the pole-ward advance of the Subtropical High resulting in increased precipitation intensity due to dynamically strengthened on-shore vapor transport, likely delivered in more intense atmospheric rivers^22^. This, together with the thermodynamic enhancement of vapor transport due to substantial warming^17^, bolsters precipitation extremes, while the frequency of mid-latitude cyclones delivering low- medium-intensity precipitation changes little.

CMIP5 GCMs are for the most part too coarsely resolved to adequately represent California’s topography; we, therefore, suspect that precipitation due specifically to ARs, being strongly orographic, is underestimated in GCMs. Thus we need downscaling to better resolve AR-related precipitation and other heavy precipitation events to better quantify future precipitation trends in orographically complex California. Here we only investigated winter (DJF) precipitation changes. However, refs 24 and 25 found that in California, projected precipitation differed between winter (somewhat wetter) and the shoulder seasons of fall and especially spring (drier in late 21st Century).

A general conclusion that applies to all Mediterranean regions is that a decrease in the frequency of daily precipitation events, combined with an increase in the amount of precipitation delivered in relatively rare heavy events, yields greater year-to-year variability in total precipitation^5, 26^, i.e. water resources become more volatile.

Results presented above, however, suggest rather different implications of global warming for drought and flood risk with different impacts on water resources, ecosystems and human health in MedClim regions of the northern and southern hemispheres. This means that different adaptation strategies will be required particularly for California compared to all other MedClim regimes.

## Methods

### Data

We use daily precipitation, sea level pressure, and surface temperature outputs from a subset of 30 climate models (all models with the required data that were available at the time of analysis) and integrated water vapor transport from 22 climate models (i.e., the models that have wind and specific humidity data available at the vertical pressure level at the time of analysis; ref. 17) that participated in the CMIP5 project (available at http://pcmdi9.llnl.gov/; ref. 27). The list of the models used can be found in the online supporting material.

The response of anthropogenic warming is evaluated as the difference between the 30-year period at the end of the century (2060–2089) projected under the RCP 8.5 emission scenario and it’s counterpart (1960–1989) in the historical simulations. The historical simulations were forced with observed long-lived greenhouse gases, solar radiation, and volcanic aerosols for the observational period, whereas the RCP 8.5 simulations were forced with an increase in the atmospheric concentration of CO_2_ to 950 ppm by the year 2100. For consistency, all modeled and observed variables considered were remapped to a common 2° × 2° latitude-longitude grid using bilinear interpolation before analysis.

### Definition of Mediterranean-climate regions

We define MedClim regions following the Köppen-Geiger climate classification scheme as having cool, damp winters and hot, dry summers, applying thresholds for seasonal temperature and precipitation at each grid (we direct readers to ref. 11 for details). The 50-year period of 1950–2000 monthly climatological observed temperature from the Global Historical Climatology Network version 2 and the Climate Anomaly Monitoring System (GHCN CAMS; ref. 28) and precipitation from the Global Precipitation Climatology Centre (GPCC; ref. 29) were used to identify the Mediterranean-climate regions on a 2° × 2° latitude-longitude grid. The five Mediterranean-climate regions around the globe defined by this scheme are indicated by stippling on Fig. 1; they include the Mediterranean basin (MED: max latitude and longitude extent, 30°–45° N, 30° W–60° E), California (CAL: 30°–42°N, 127°–115°W), South Africa (SAF: 33°–37°S, 15°–20°E), South America (SAA: 30°–45°S, 75°–70°W), and Australia (AUS: 32°–42°N, 110°–142°E).

### Definition of dry day

GCMs typically overestimate the frequency of days with small precipitation accumulation^12, 30^ while observations do not report precipitation below a certain threshold. For consistency between models and observations we select a threshold of 1 mm/day, below which both observations and models, re-gridded to the same 2 × 2° grid, are considered to have a dry day (we direct readers to ref. 5 for choice of threshold used and further details).

### Graphic software

All maps were produced using NCAR Command Language Version 6.3.0 (NCL).

## Electronic supplementary material

Supplementary information
