# Supplementary material for: Precipitation in a warming world: Assessing projected hydro-climate changes in California and other Mediterranean climate regions
Source: Sci Rep. 2017 Sep 7;7:10783. doi: 10.1038/s41598-017-11285-y (PMC5589768; doi:10.1038/s41598-017-11285-y)
Supplement: Supplementary file 1 — Supplementary information [file 41598_2017_11285_MOESM1_ESM.pdf]

Precipitation in a warming world: Assessing projected hydro-climate changes in California and other Mediterranean climate regions

Suraj D Polade[1], Alexander Gershunov[1], Daniel R. Cayan[1], Michael D. Dettinger[2], and David W. Pierce[1]

Supplementary information

[1]Climate, Atmospheric Science and Physical Oceanography (CASPO), Scripps Institution of Oceanography, La Jolla, CA, USA
[2]United States Geologic Survey, Carson City, Nevada, USA

S1. List of the coupled atmosphere-ocean climate models used in this study

The 30 CMIP5 models used are **ACCESS1-0**, **ACCESS1-3**, **BCC-CSM1-1**, **BCC-CSM1-1-M**, **BNU-ESM**, **CanESM2**, CCSM4, CESM1-BGC, CMCC-CESM, CMCC-CM, CMCC-CMS, **CNRM-CM5**, **CSIRO-Mk3-6-0**, EC-EARTH, **FGOALS-g2**, **GFDL-CM3**, **GFDL-ESM2G**, GFDL-ESM2M, **HadGEM2-CC**, HadGEM2-ES, **INM-CM4**, **IPSL-CM5A-LR**, **IPSL-CM5B-LR**, **MIROC-ESM**, **MIROC-ESM-CHEM**, MIROC5, **MPI-ESM-LR**, **MPI-ESM-MR**, **MRI-CGCM3**, **NorESM1-M**. Model selection is primarily done on the basis of availability of daily data for all the variables considered for the analysis period. Bold letter indicates models providing wind and moisture data needed to compute integrated vapor transport (IVT).

S2. Seasonal cycle of precipitation over mediterranean-climate regions and mean winter precipitation changes

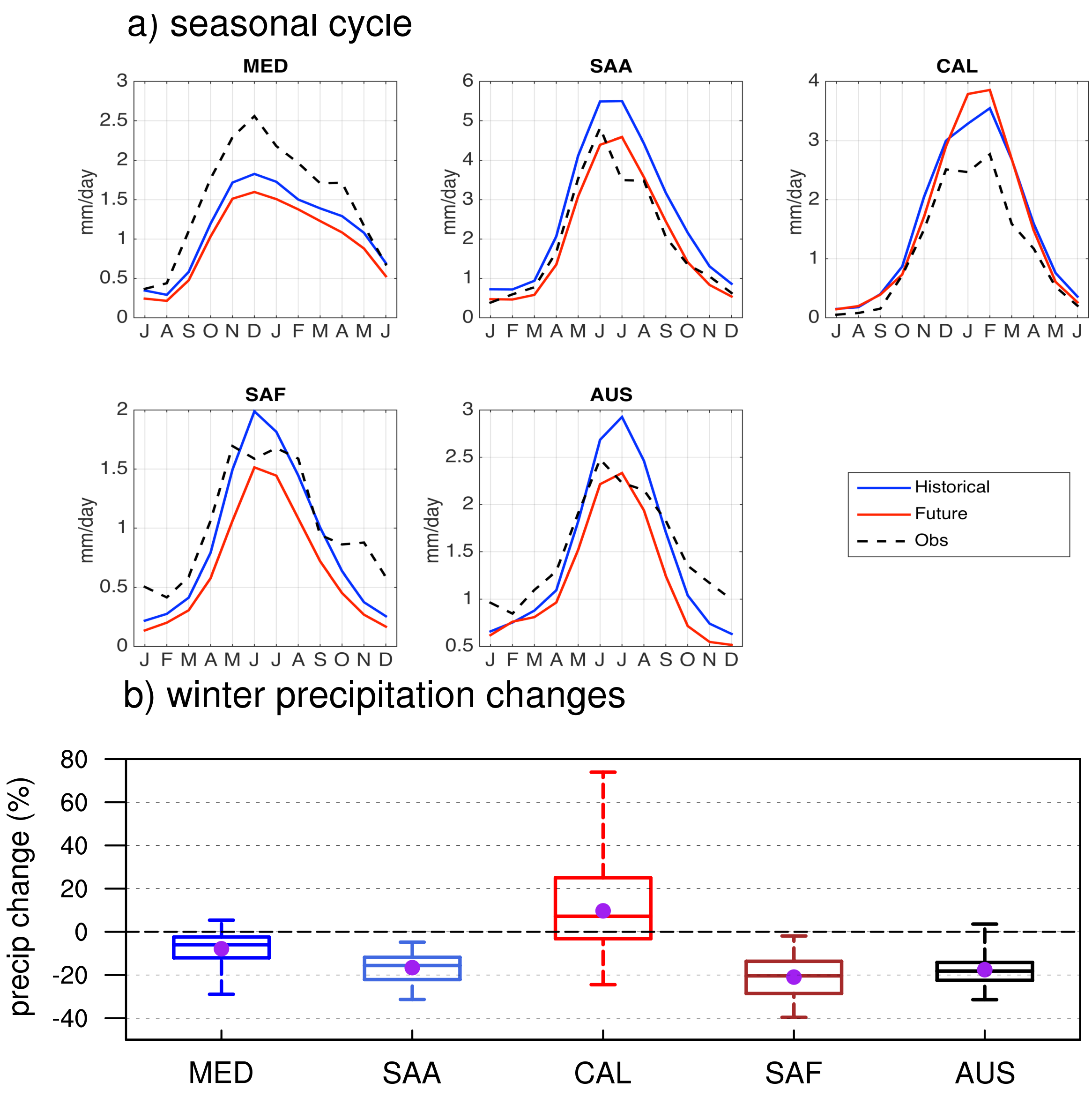

Figure S1: GPCP observed and CMIP5 multi-model ensemble mean precipitation a), seasonal cycle and CMIP5 winter precipitation changes b) area average over the mediterranean-climate grids (stippled region shown in Fig. 1a) by 2060-2089, relative to the historical period 1960–1989, using the RCP8.5 forcing scenario over the five global mediterranean-climate regions. Multi-model ensemble averages are shown by circles and medians indicated by horizontal lines inside the box.

Model realism and resolution are explored in Figures S2 – S3 below in terms of its effect on model performance.

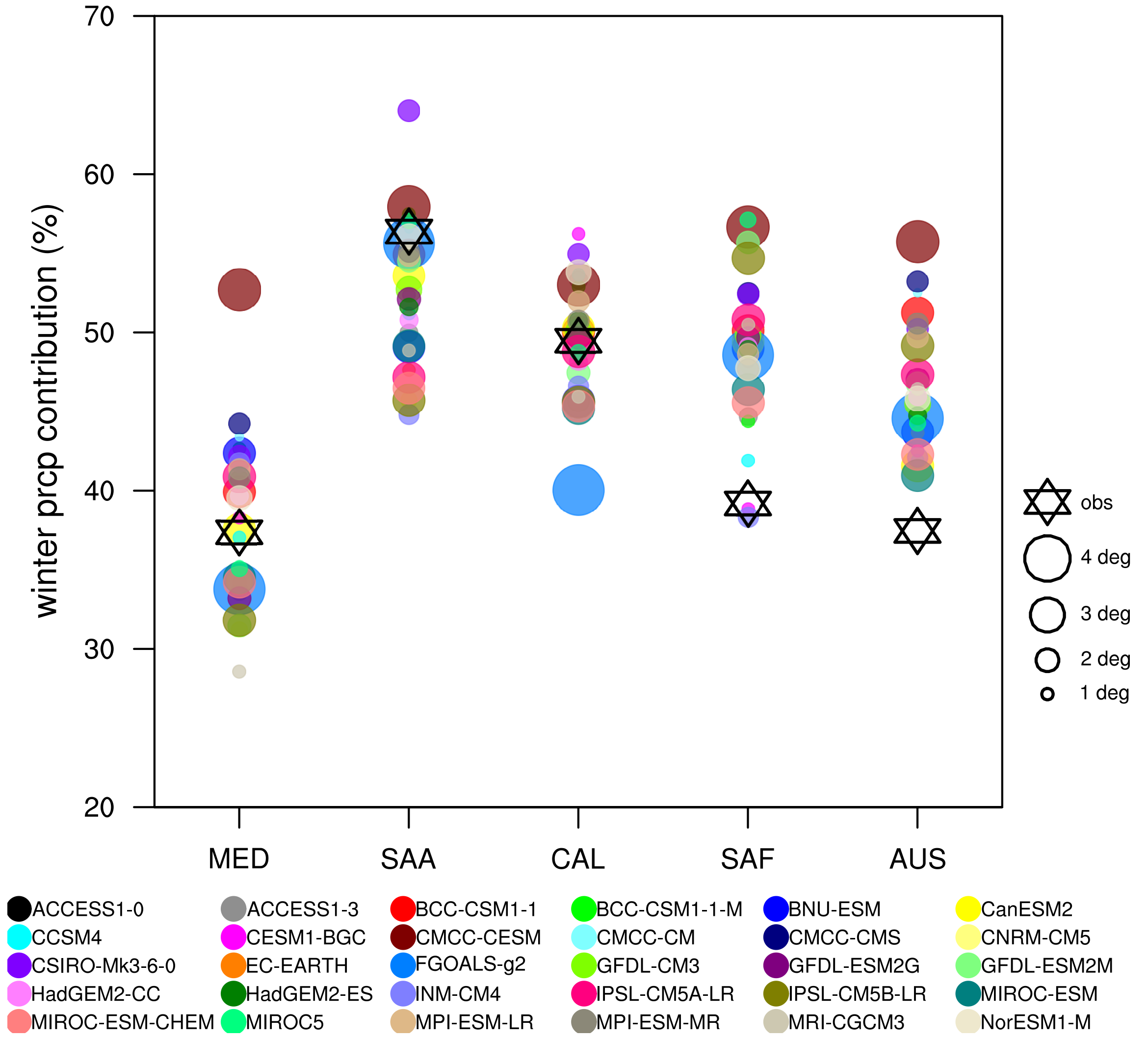

Figure S2. Winter contribution to the annual total precipitation (y-axis) by region (x-axis) in observations (star) and models (colors). Model resolution is denoted by the size of the circles. This result is based on the historical period (1960-1989).

The contribution of winter (DJF) precipitation to the annual total is somewhat better reproduced by the models over the MED and CAL, but poorly over SAF and AUS. A

close examination of Figure S2 suggests that GCM ability to skillfully reproduce this

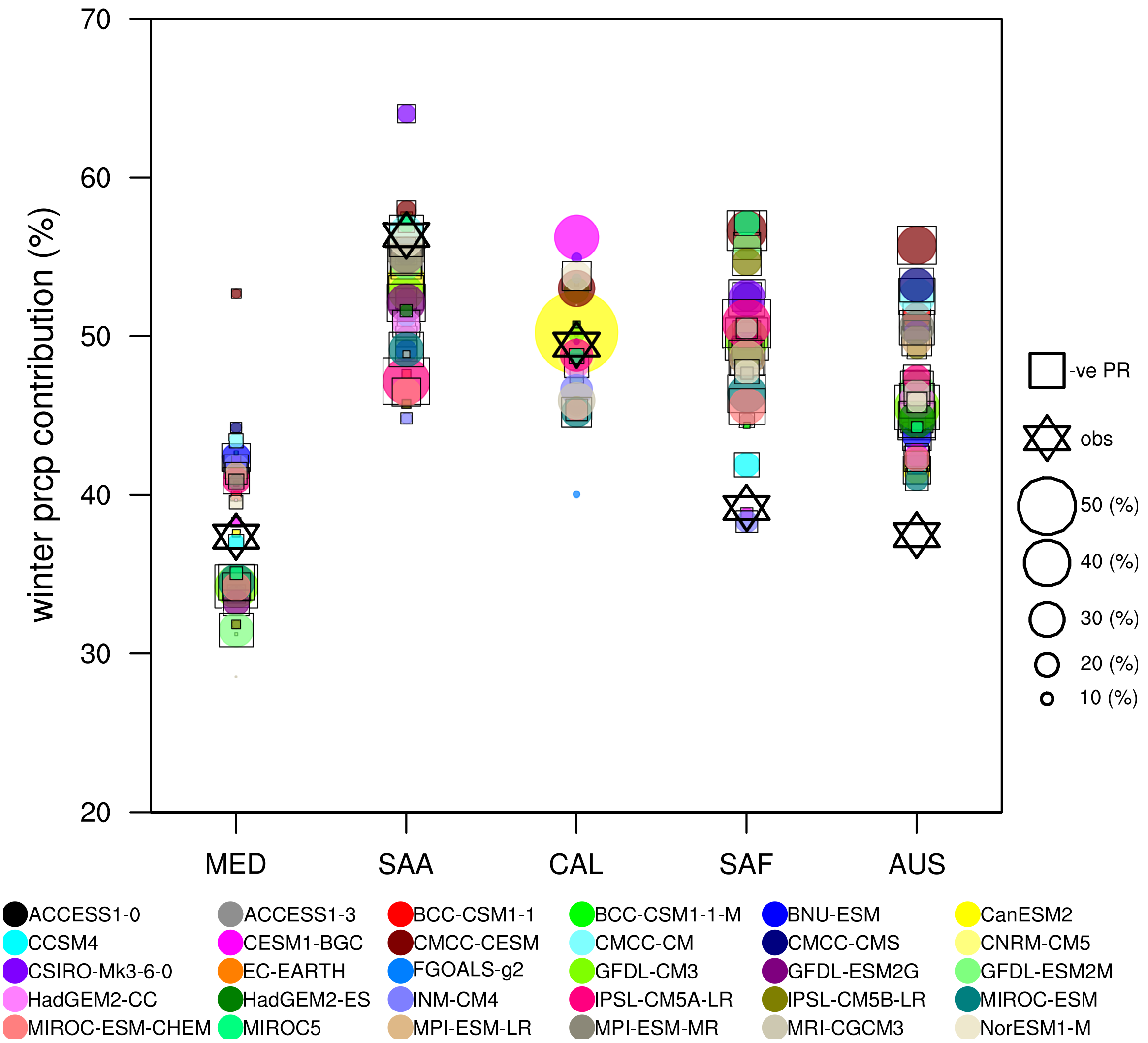

essential feature of Mediterranean climate is not a function of GCM resolution

Figure S3. Winter precipitation contribution to annual (y-axis) versus projected changes (circle size) by region (x-axis) versus. Negative changes (drying) are denoted by boxed circles. Regular circles denote positive change.

Realism in simulating winter contribution to total precipitation does not systematically affect how much change is projected or even the sign of the change in the case of CAL.

S3. Wintertime changes in precipitation versus mean sea level pressure

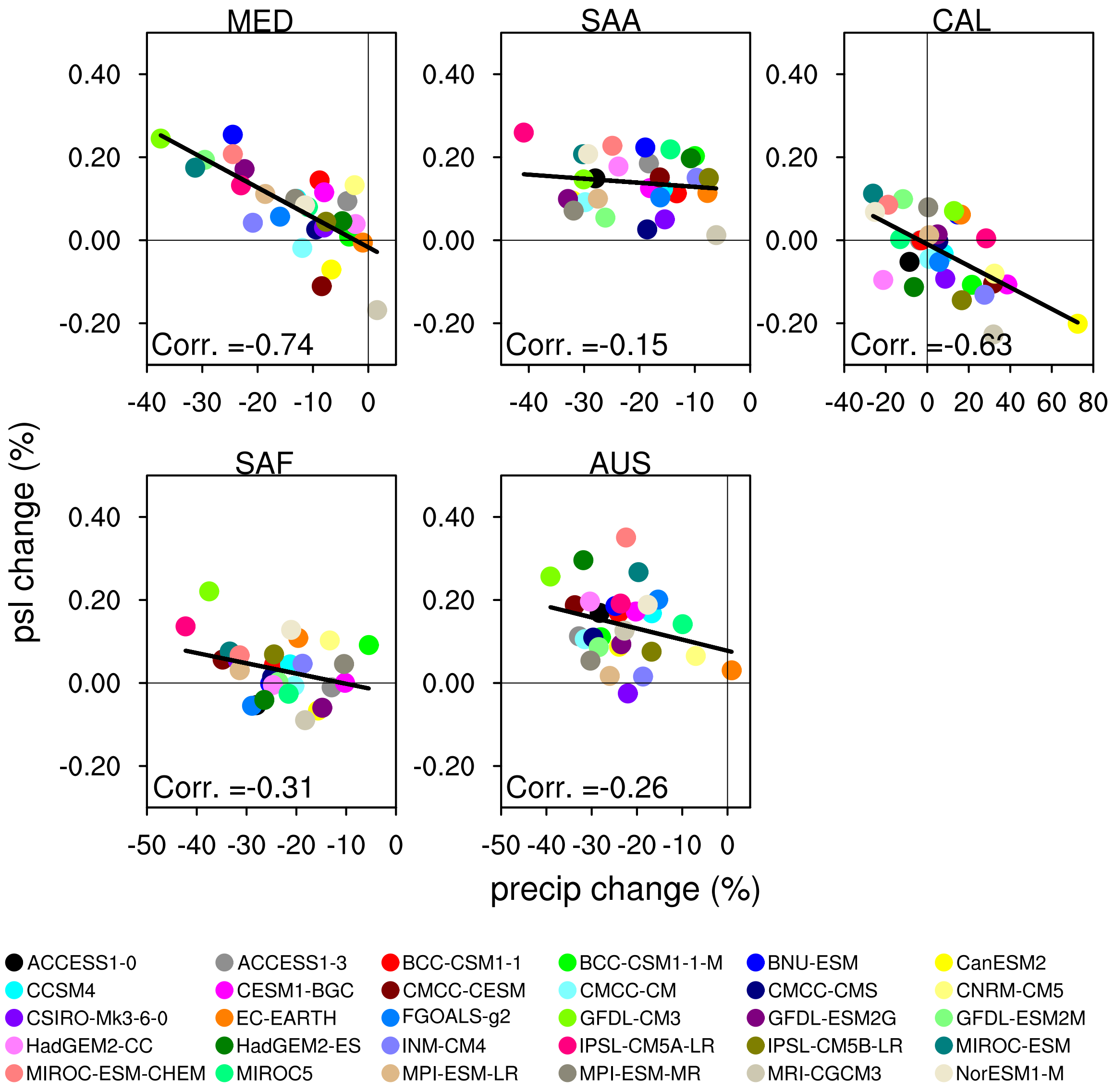

Figure S4: CMIP5 winter changes in precipitation versus mean sea level pressure average over the mediterranean-climate grids (stippled region shown in Fig. Sa1) by 2060-2089, relative to the historical period 1960–1989, using the RCP8.5 forcing scenario over the five global mediterranean-climate regions. Black line indicates the regression line (and correlation, Corr) corresponding to all CMIP5 models.
